# Memory for own actions in parrots

**DOI:** 10.1038/s41598-022-25199-x

**Published:** 2022-11-29

**Authors:** Sara Torres Ortiz, Simeon Q. Smeele, Juliette Champenois, Auguste M. P. von Bayern

**Affiliations:** 1 Max Planck Institute for Biological Intelligence, in Foundation, Seewiesen Eberhard-Gwinner-Strasse, 82319 Starnberg, Germany; 2 Max-Planck Comparative Cognition Research Station, Loro Parque Fundación, Av. Loro Parque, 38400 Puerto de la Cruz, Tenerife Spain; 3 Max Planck Institute of Animal Behavior, Am Obstberg 1, 78315 Radolfzell Am Bodensee, Germany; 4 Max Planck Institute for Evolutionary Anthropology, Deutscher Platz 6, 04103 Leipzig, Germany; 5 Department of Biology, University of Konstanz, Konstanz, Germany

**Keywords:** Ecology, Evolution, Psychology, Zoology

## Abstract

The ability to recall one’s past actions is a crucial prerequisite for mental self-representation and episodic memory. We studied whether blue-throated macaws, a social macaw species, can remember their previous actions. The parrots were trained to repeat four previously learned actions upon command. Test sessions included repeat trials, double repeat trials and trials without repeat intermixed to test if the parrots repeated correctly, only when requested and not relying on a representation of the last behavioral command. Following their success, the parrots also received sessions with increasing time delays preceding the repeat command and successfully mastered 12–15 s delays. The parrots successfully transferred the repeat command spontaneously at first trial to three newly trained behaviors they had never repeated before, and also succeeded in a second trial intermixed with already trained actions (untrained repeat tests). This corroborates that successful repeating is not just an artifact of intense training but that blue-throated macaws can transfer the abstract “repeat rule” to untrained action. It also implies that an important aspect of self-representation has evolved in this avian group and might be adaptive, which is consistent with the complex socio-ecological environment of parrots and previous demonstrations of their complex cognition.

## Introduction

Most of human everyday memories involve past actions, i.e., memories of what oneself or others did in the past^1^. Such memories of past events involving past actions shape current behavior, as well as guide future behavior^2^. Yet, most memory studies on humans and non-human animals are based on remembering passively presented stimuli, without overtly involving and often even restricting actions^1,3,4^. Besides involving overt motor behavior, actions are also different from the traditional passive stimulus presentation approach because they are self-performed^1,3–5^. Given that the definition of human episodic memory^6,7^ is the “memory for personally experienced events” and that self-performed actions are per se “personally experienced”, memory of self-performed actions should be considered episodic accordingly. Being able to recall and represent one's own past actions is at the same time considered as of the main prerequisites or main “building blocks” for the ability to represent the self^8–10^. It has been speculated that it may have evolved early in mammalian evolution and could be widespread phylogenetically, since it has recently been documented in domestic dogs^3,8,11^ (*Canis lupus familiaris*), as well as dolphins, primates and pinnipeds^12–14^. Given that self-representation in turn is a prerequisite for human episodic memory^15^, this applies to remembering one’s own past actions inevitably. As self-awareness of one's own personal experiences is not clearly proven in animals, the analogous memory processes in animals are often referred to as episodic-like memory^15,16^. To date, several studies have reported evidence for episodic-like memory in a few animal species although most of them remain debated. For example Clayton and colleagues^17^ claimed the first evidence for episodic-like memory showing that scrub jays (*Aphelocoma californica*) are able to remember where and when they have cached a particular food item, and claimed it, could imply the ability of episodic memory for the species. However, it was argued that food caching and retrieval behavior is very likely genetically fixed, and the paradigm used failed to provide evidence of conscious (‘autonoetic’) components of episodic memory possible in a scrub jay^18^, thus, the ability was called episodic-like memory^19–21^.

Testing an animal’s ability to repeat its own previous behavior, i.e., the “repeat paradigm”, has been put forward as a method to determine animals’ memory for their own actions^12,22^ and recently has been proposed as a method of testing episodic-like memory in two studies on dogs^8,11^. The methodology has great potential as a comparative test for episodic-like memory content in animals, but the prerequisites for truly episodic-like memory retrieval rely on the assumption that the animals must not expect to be asked about the past event^23^. The unexpectedness of the test rules out a prepared behavioral response (i.e., the animal expects the trial and gets ready to offer the correct behavioral response rather than retrieving the past event from their memory) from the animal, instead of an episodic content retrieval^8,24^.

To date, the “repeat paradigm” has been employed in pinnipeds, pigtail macaques (*Macaca nemestrina*), dogs and dolphins (*Tursiops truncatus*)^8,11–14^, i.e., four mammalian taxa. Even though all tested species learned to repeat their previous behavior on a range of behaviors they could perform on command, the extent to which the different species relied on the underlying repeat concept, i.e., “repeat your last action” remains unclear. Little detail was given for the monkeys, dogs and dolphins regarding the training procedure and previous experience, making comparisons between species difficult. In any case, dolphins and dogs were reported to generalize the repeat command to novel behaviors^8,11,12^ and macaques were found to fail^13^ while pinnipeds were not tested^14^. Concerning the duration of memory retention, the species differed substantially, but given the methodological differences between the studies, one cannot directly compare these results and draw reliable conclusions from them.

In order to gain deeper insights into the phylogenetic distribution of the ability to recall self-performed actions, it is necessary to broaden the comparison to species outside of the mammalian clade. Parrots, a diverse avian order, appear an interesting first model group for investigating memory for own actions because together with dolphins, primates and corvids they stand out among vertebrates in terms of both their relative brain size^25^ and neuron density^26,27^ and are considered on a par with great apes in terms of complex cognition^25^. Parrots exhibit high social complexity and long-term monogamy^28^ and share several socio-ecological and cognitive traits with dolphins and primates^29–31^. Various memory studies have been conducted in different avian species, ranging from corvids to pigeons^16,32–34^ but to date, memory for own actions has not been investigated in birds. In parrots, no study has focused on memory specifically, although it seems equally adaptive for them than it has been suggested to be for corvids^35–37^. Albeit, many cognitive abilities that have been studied in parrots involve short-term and possibly other types of memory. For example, previous work on African grey parrots *(Psittacus erithacus)* has shown that they can categorize objects, recognize similarity and point out missing information, all of which requires short-term memory^38^. For instance, in order to show appropriate social behavior, individuals may have to remember specific social events (in terms of what happened where and when and who was involved) that could lead to, for example, sudden changes in the hierarchy. According to Clayton et al.^16^ monitoring the interactions of conspecifics and remembering specific social events is crucial for an updated knowledge of the relationships and social dynamics between one’s group members. The type of memory necessary to perform these functions fits the behavioral criteria of episodic memory. Such a memory of social interactions would of course also include events of one’s own interactions with conspecifics. A good example is a study by Emery and Clayton^39^ conducted with scrub-jays where one group of birds experienced stealing other birds’ caches whereas a second group lacked this experience. Only the group that had experienced being a thief re-cached their food after having been observed by other individuals^35,39^. If convergent trends in the evolution of sociality and cognition hold, we should therefore see robust memory for one’s own past actions in parrots, another avian taxon.

The objective of the current study was to extend our understanding of memory for own actions in mammalian taxa to a distantly related avian model, and to examine the parrots’ concept learning ability. We tested blue-throated macaws (*Ara glaucogularis*), a social, large-brained species, endemic to Bolivian Savannah type habitats^40^ that had been trained to perform four different arbitrary behaviors upon specific commands (hand signals). After the training of the specific actions, they were trained to respond to the repeat command reliably, i.e., repeating one out of the four actions they had previously performed upon command when requested to “repeat” (details of their training procedure are provided in Supplementary materials). Depending on the experimental condition, they had to repeat straight after the performed action, or after an increasing time delay. Extending previous studies, we also tested the parrot’s ability to generalize the repeat rule to novel behaviors. Considering the results from the mammalian studies^8,11,13,14,22^ and given that parrots share similar socio-ecological environments^30,41^ and comparable cognitive abilities^41–45^, we hypothesized that the macaws will be able to learn the abstract rule of repeating their own previous action, to remember self-performed behavior for delays comparable to those of the mammals tested, and to transfer the repeat command to novel actions.

## Results

### Repeat test

#### Single repeat trials

**Figure 1:**
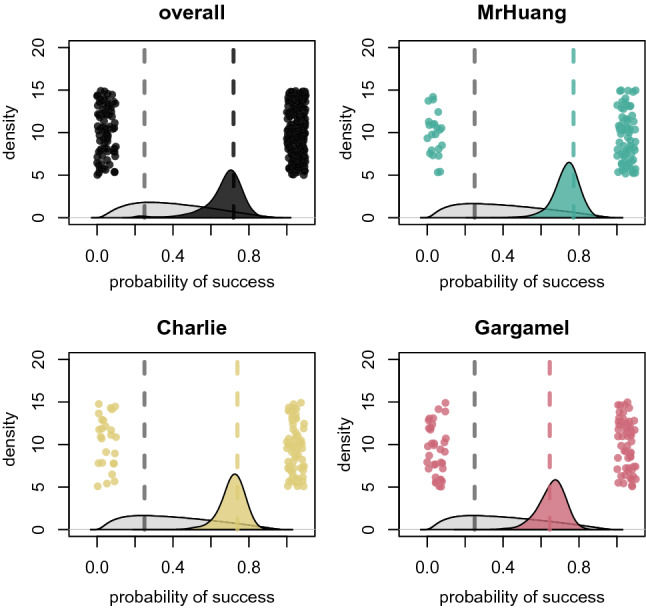
Performance in the single repeat trials. Gray density plots show the prior centered around chance-level (gray dashed line). Colored density plots show the posterior distributions for the average performance. Dashed colored lines are the means. Dots are outcomes per trial.

**Figure 2:**
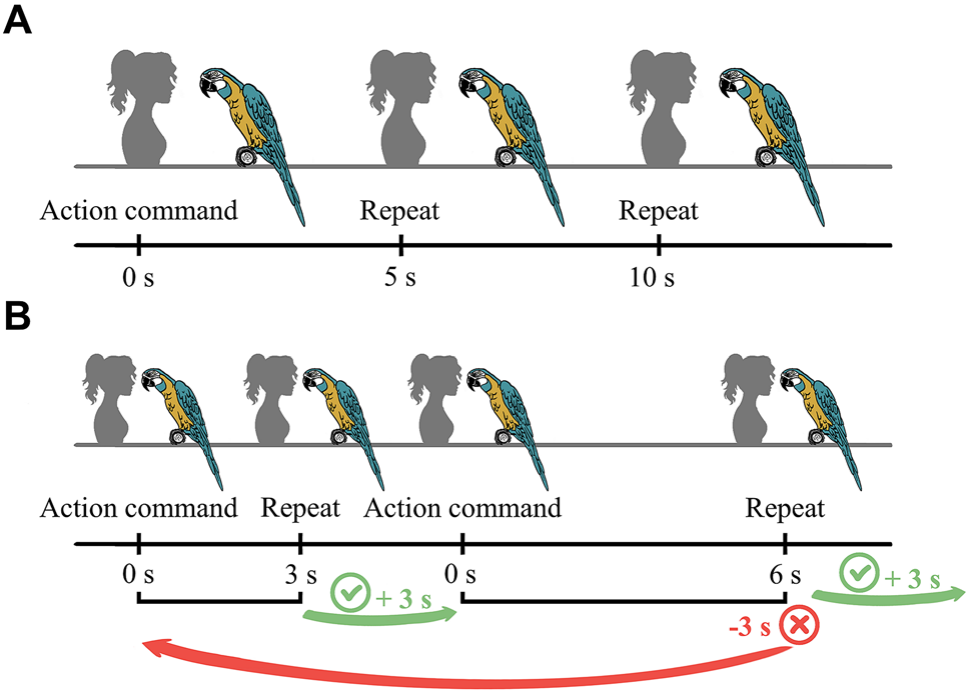
(**A**) Illustration for the double-repeat trial procedure and the delays between the different commands. (**B**) Delayed repeat test procedure. If the parrot repeated correctly, the delay increased three seconds in the next trial, otherwise, the delay decreased three seconds (artwork by Sara Torres Ortiz).

All parrots showed overall performance well above chance level across all four behaviors, about 75% with a chance level of 25% (see Fig. 1; see Table SP1) in the single repeat trials (Fig. 1). There were no differences between the individuals (sigma: 0.46, 89% PI: 0.04–1.31) and the behavior to be repeated had little effect on the performance except for ‘Head shake’, which all parrots were less likely to successfully repeat (sigma: 0.45, 89% PI: 0.09–0.95). The time between the command for the action and the repeat command was five seconds (Fig. 2A).

#### Double repeat trials

**Figure 3:**
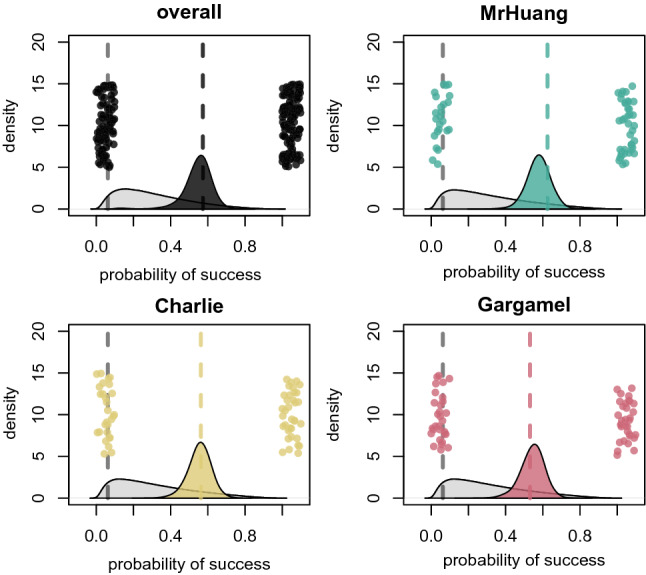
Performance in the double repeat trials. Gray density plots show the prior centered around chance-level (gray dashed line). Colored density plots show the posterior distributions for the average performance. Dashed colored lines are the means. Dots are outcomes per trial.

In the double repeat trials, all three birds also performed well above chance level across all four behaviors with an overall performance of 60% correct, with a chance level of 6.25% (Fig. 3). There were no significant differences between individuals (sigma: 0.47, 89% PI: 0.03–1.4) and behaviors (sigma: 0.89, 89% PI: 0.19–1.7). The time between the action command and the second repeat was 10 s (Fig. 2B).

#### Delayed repeat test

**Figure 4:**
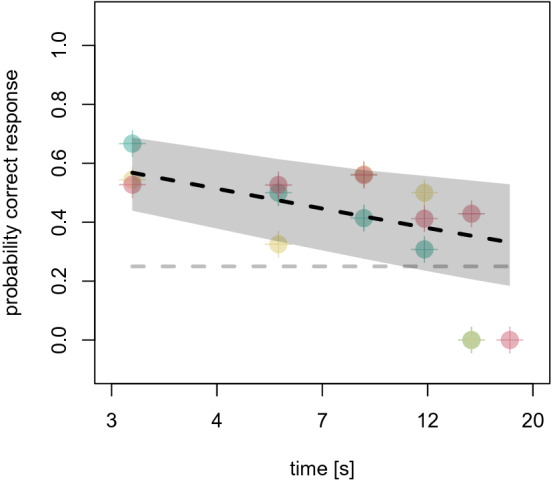
Performance in the delayed repeat test. Colored dots are average performance per delay and individual (green—Mr Huang, yellow—Charlie, red—Gargamel). Dashed line is the predicted performance and gray shaded area the 89% PI.

The parrots were able to perform above chance level for delays up to 12–15 s (see Fig. 4). There were no significant differences between the three individuals (sigma: 0.48, 89% PI: 0.03–1.37) and behaviors (sigma: 0.55, 89% PI: 0.04–1.30). The performance in the delayed (single) repeat test with a corresponding delay of 10 s was 41% (0.89 PI: 26–57%; see Fig. 4).

#### Novel behavior repeat test

All three parrots were able to repeat newly learned behaviors spontaneously in the first and in a second test trial, which were intermixed with already repeated behaviors. Table 1 summarizes the animals’ performance in repeating novel behaviors. “Success” refers to successful repeating in both test trials. All three parrots succeeded repeating the behavior “vocalization”. Concerning the second novel behavior, “lift left leg”, only Charlie lifted the correct same leg when asked to repeat, while Mr Huang and Gargamel lifted the right leg. The last tested novel behavior, “Move ring into lid”, was mastered spontaneously by Mr Huang and Gargamel, they both moved the ring into the lid. Charlie instead lifted the left leg during the session, when asked to repeat the “Move ring into lid”, so performed the previous learned behavior. Thus, all individuals repeated two out of three novel behaviors in their first and second trial without training.

**Table 1:** Summary of the results of the novel behavior repeat test.

Animal ID	Behavior	Testing date	Result
Charlie	**Vocalization**	28/06/2021	**Success**
	**Lift left leg**	15/08/2021	**Success**
	Move ring into lid	16/09/2021	Lifted leg
Mr Huang	**Vocalization**	30/06/2021	**Success**
	Lift left leg	15/08/2021	Lifted right leg
	**Move ring into lid**	29/09/2021	**Success**
Gargamel	**Vocalization**	05/09/2021	**Success**
	Lift left leg	11/09/2021	Lifted right leg
	**Move ring into lid**	04/10/2021	**Success**

## Discussion

The blue-throated macaws tested in this study could recall their own previous actions well above chance levels, providing the first evidence for mental representation and memorization of their own actions in an avian taxon. The overall (single) repeat performance of parrots was similar to those of mammals. Fugazza et al.^8^ hypothesized that one of the building blocks of the supposedly complex ability to represent the self might be the capacity to mentally represent one’s own behavior. Our study shows that this ability is also present in parrots and therefore suggests it has evolved in birds too. This is an interesting finding considering the evolutionary distance between mammals and birds. Birds also have differently structured brains compared to mammals although their forebrain (nido- and mesopallium) represents a homologous structure to the mammalian neocortex^28,46–48^. Like the neocortex, this telencephalic structure fulfills higher cognitive functions even if it lacks the cortex-typical lamination and is structured differently^49^. Even though parrots’ brains may be small in absolute size, their relative brain sizes stand out amongst all vertebrates like otherwise only those of corvids, primates and dolphins^31^. Additionally, parrots exhibit an astonishing neuronal density in their pallium with neuron numbers exceeding those of primate species^27^. It may be the large absolute numbers of telencephalic neurons in their pallium that explains their advanced behavioral and cognitive complexity^27,28,50^. Together, the architectural differences in the brains of mammals and birds and the great evolutionary distance indicate that the ability to remember their own actions is likely a result of convergent evolution.

The finding that the birds performed above chance level and similarly well in single and double repeat trials suggests that the birds relied on their memory of their last own previous action rather than the last command (i.e., the previous hand signal) they received. The interval between the command for a behavioral action and the second repeat command was on average 10 s. If the parrots were remembering the hand gesture, their performance of the second repeat should have been similar to the performance of the delayed condition at 10 s (Fig. 2A,B). However, the parrots’ performance in the delayed (single) repeat test with a corresponding delay of 10 s was 20% lower compared to their performance in the double repeat. It is therefore unlikely that the parrots remembered the last command rather than responding to the repeat command. The better performance at double repeating compared to remembering following a delay of 10 s suggests that the parrots remembered their last action, which was performed only 5 s earlier, rather than the last command.

Our other objective was to examine whether the parrots had actually abstracted the underlying rule of repeating their last performed action, which can be considered as an abstract concept^11^. The parrots had been trained with four behaviors only before it was tested whether they had learned the “repeat” rule and could transfer it to novel actions they had not repeated before (novel behavior repeat test). Their spontaneous success shows that indeed they had generalized the repeat rule. The fact that parrots learned this abstract concept after only being trained for four actions is remarkable. By comparison, pigeons, monkeys, parrots and corvids needed thousands of trials to learn the same-different concept required to succeed in match-to-sample tests^51,52^. Similarly, sea lions need over two thousand trials to form equivalence relations, where the animal needs to spontaneously transferred the relationship on a set that is reflexive (A ~ A), symmetric (if A ~ B, then B ~ A) and transitive (if A ~ B and B ~ C, then A ~ C)^53,54^. In general, a conceptual ability provides great efficiency to learning^55^. Fast concept learning and generalization may be adaptive under many circumstances as it allows the individual to apply prior learning to a new environment avoiding the costs and risks associated with new trial-and-error learning^55^. Why the macaws tested in this study showed such a rapid concept learning ability, needs to be solidified by further direct comparisons of different taxa in the same tasks and remains speculation until it is investigated further. Maybe parrots need to adapt fast to novel environments or social constellations but the same is likely true for primates and pinnipeds^14,56^.

The other important conclusion one can draw from the parrots’ spontaneous transfer ability is that they could recall their own previous behavior without being trained to do so. Given that the repeat command was never associated with novel actions, we can assume that they did not expect the repeat command after the novel action in the experimental test. According to Fugazza et al.^8^, the unexpectedness of the test rules out a “prepared behavioral response” explanation and suggests accidental encoding of the memory^8^. In our experiment, it can be assumed that repeating the novel behavior is unexpected by the parrots. Firstly, because the novel behaviors were never asked to be repeated, and secondly, because of the long break between the delayed repeat experiment and the novel behavior repeat test (~ 45 days). Accordingly, the parrot’s success in retrieving the memory of the novel behavior would have to be explained by episodic-like memory. Of the four previously tested mammalian taxa, only dogs have been tested for accidental encoding (albeit with substantially longer time scales)^8^ and only dolphins and dogs were shown to generalize the repeat command to new behaviors^8,11,12^. The pinniped species were not tested in this respect and the macaques were not able to transfer the repeat rule having been trained with three behaviors only^13,14^. The dolphins^12,22^ transferred the repeat rule to new behaviors but already knew the repeat command for a long time and there is no information on how many behaviors they required to learn the repeat rule before they were capable of generalizing. Both dog studies^8,11^ reported that the subjects were able to transfer the repeat rule after they had trained the repeat command on six to seven behaviors. Although the data on the different species are not directly comparable due to the differences in methodology, particularly concerning the previous training exposure, our results suggest that parrots have a capacity to generalize abstract rules comparable to big-brained mammals as dolphins in the same paradigm. Domestic dogs which also generalized well, may represent a special case because of domestication and enculturation^57,58^. For instance, studies on wolves raised by humans and dogs with little human contact showed that dogs were still better at reading human communicative gestures, suggesting that artificial selection for a set of social-cognitive abilities enhanced their responsiveness to human communicative signals^59^. Similar findings have been reported in birds as well, e.g., artificial selection negatively affecting spatial learning ability in white leghorn chicken compared to their ancestor the red jungle fowl^60^. In any case, such effects of artificial selection and enculturation should be considered in phylogenetic comparisons of cognitive abilities and may explain why dogs performed so well in this task.

Concerning the duration of memory retention, our results show that parrots could remember their own behavior for up to 12–15 s. The retention interval is similar to those previously reported in the wide variety of species tested in Delayed-Match-to-Sample, a common paradigm to test animals’ working memory^4,61^. Our results are also quite similar to those reported in pinnipeds, macaques and dolphins tested previously in this paradigm^13,14,22^ but dogs exhibited longer retention intervals. These differences can be explained by methodological differences. In one of the dog studies, the delay intervals were fixed with 10 and 30 s, in our case, if the parrots made a mistake, the interval would decrease 3 s so that the parrots were never exposed to such long intervals^11^. In the second dog study, the trial for the delay condition only contained a single action that had to be repeated after 1 h^8^. For parrots, each experimental session had 16 trials, which adds noise into their memory system increasing the attention needed to perform correctly^8^. As parrots paralleled other mammal species in performance for single and double repeats, it is likely that the reported differences are a result of different methodology. Unfortunately, details of the training methodology of the previous studies are lacking. Replicating the previous pinniped study in this respect, the parrots were not trained for expecting the delays before entering the delayed repeat test, so it was tested how well they remembered when not actively trying to remember. Concerning the dogs, in contrast, no details are given as to whether the delay was learned with training steps gradually increasing in duration, or whether the animals were directly tested at their longest retention durations in an unexpected manner. The same challenge is present when comparing results of Delayed-Match-to-Sample tasks, as the amount of training is not always reported in sufficient detail, or at all, making species comparisons in memory performance very challenging^4^.

The incredibly complex brains of humans and varied faculties are likely to have evolved from simpler prototypes of our ancestors^62^. Paul Cisek developed the theory of “phylogenetic refinement” where behaviors and brain structures are the consequence of evolutionary refinement from more basic building blocks^63^. One may see the parrots’ ability to represent and remember their own actions as an important building block of the ability to represent “the self”^8^. In order to experience one's self in the past one would need an awareness of self also in the present time^1^. Thus, the parrots could be considered have evolved a prerequisite of episodic-like memory. Fugazza and colleagues^8,24^ state that previous studies can be explained by a “prepared behavioral response” by the subjects as they expected the repeat command to be given. We argue that the repetition of a novel action can be considered as accidental encoding in our study given that the parrots had never been asked to repeat those actions before. Additionally, the test of the first untrained action for the novel repeat test took place following a ~ 45-day break after the delayed repeat test had been completed, so that the parrots were not in a repeat testing routine anymore.

Like most parrots, blue-throated macaws are very social, show fission–fusion dynamics with temporally changing flock composition (e.g., for foraging or during certain developmental phases^40^) and live in long-term monogamous pairs throughout their lives^64^, all of which likely has selected for socio-cognitive adaptations and even larger relative brain sizes^26,50^. Being able to remember one’s own behavior may be equally adaptive for species living in complex societies^31,65^ than remembering the behavior and interactions of third-parties^50,66,67^. Concerning parrots, remembering one’s own past action might be particularly important for coordinating cooperative behaviors with one’s mate (e.g., when jointly rearing offspring^28^) or group members (e.g., when foraging individuals of a flock leave to join other groups^29^) for which parrots exhibit the cognitive and motivational basis^42,44,68–70^. Parrots also have been shown to display capacity for reciprocity, which may require long-term episodic memories of own and others’ behavior^44,66,69^ for the reciprocal sharing of resources.

In summary, blue-throated macaws were proficient in learning and generalizing the abstract concept of “repeat my most recent action”. Our results show that parrots are capable of reporting their own previous actions upon command. They therefore exhibit a pre-requisite for self-representation and for episodic-like memory previously only shown in social, large-brained, domesticated or enculturated mammals. Given the evolutionary distance between mammals and birds^31^ with a common ancestor around 297 mya^31^, this ability is likely to constitute an example of convergent or independent evolution, due to similar selection pressures^28,30,31,71^. Our study provides new insights into the independent evolution of functionally equivalent building blocks of self-representation and episodic-like memory in distantly related taxa^10,20^.

## Materials and methods

### Subjects and housing

Three adult male blue-throated macaws (*Ara glaucogularis*) (between 10 and 12 years old) were trained and subsequently tested. Details on age and sex of the individuals are specified in Table SP2. The training of the behavioral commands and repeat commands are described in the Supplementary materials.

The birds were housed in the Comparative Cognition Research Station, inside Loro Parque zoo, in Tenerife (Spain). Loro Parque Foundation staff hand-raised and group-reared all the parrots used in this study (more details on housing and diet are described in Supplementary materials). Water was provided at libitum and the parrots were fed twice a day. All parrots participated voluntarily both in the training sessions and the experimental sessions. During training, sunflower seeds were used as rewards, and during testing small pieces of walnut were used.

### Experimental setup and general procedures

The birds were trained and tested individually in separate testing rooms (1.5 × 1.5 × 1.5 m) artificially lit with daylight lamps (Arcadia Zoo Bars ©), which the birds were well habituated to. During the experiment, the subject sat on a perch facing the experimenter who stood inside the test room on the opposite side wearing mirror-glass and blinded sunglasses. A second person, the assistant, also wore mirrored but see-through sunglasses and observed the experiment through a window from the neighboring room. The experimenter gave the experimental commands (Table SP1) to the parrot, but could not see its response. If the parrot responded correctly to the command, the assistant in the neighboring room gave the parrot a whistle blow as immediate conditioned reinforcer, followed by a food reward (a piece of walnut) as positive reinforcement. If the parrot did not respond correctly, the whistle was not blown, no reward was given and the next trial started following a three second pause. The experimenter gave an equal piece of walnut every time the parrot performed the correct behavior in control trials. The order of the behavioral commands and repeat commands given in each session, was pseudo-randomized and counterbalanced across birds and determined before the session. The assistant also signaled to the experimenter which command to give next by showing the behavior label on the screen of an iPad visible only for the experimenter (see Video 1).

### Testing criterion

After all animals had been trained for the four actions and the repeat command, the reliability of their response to the repeat command was tested in a 20-trial session. The command for one of the four trained behaviors was given either followed by a “repeat” command in 60% of the trials or by a command for one of the four behaviors (40%). This was implemented to prevent the animal from learning to simply repeat the first command in every new trial. The list of commands was randomized and counter-balanced. To reach criterion, the animal had to perform at least seven repeats out of 12 (58%) correctly during the session.

## Experimental conditions

### Repeat test

To test the animal’s ability to repeat their own previous behavior on command, eight experimental sessions consisting of 26 trials each were completed. A “single repeat” trial was composed of the command for one of the four trained actions followed by the repeat command. A “double repeat” trial started with the command for one of the four trained actions followed by a repeat and a second repeat command. There was no training for the double repeat trials. The “control” trials consisted of requesting one of the four trained actions, followed by the command for another one of the four trained actions. For each session four of the trials (15%) were “single repeats”, eight (30%) were “double repeats” and ten trials (38%) were “controls”. The first “repeat” of a “double repeat” trial was analyzed as a “single repeat trial” for the results.

### Delayed repeat test

To test how long the animals could remember their own previous behavior, gradually increasing delays were introduced between the behavior and the repeat command. Only single repeat trials (16 trials per session, and 6 sessions) were performed. A staircase paradigm in which a delay increased 3 s after a correct response and decreased 3 s after an incorrect response was implemented.

For the delay test, the assistant stood next to the experimenter inside the room together with the parrot. The assistant held a computer and communicated the next command to be given verbally to the experimenter (see Video 2). The list of behaviors was imported into an R session (RStudio, version 1.1.383^45^). After having entered the parrot’s response (correct or not) and the delay had passed, the computer displayed the next behavior to be requested to the assistant. The assistant then communicated it to the experimenter, The computer automatically updated the delay duration for the following trial.

### Novel behavior repeat test

To test if the parrots could generalize the repeat rule and apply it to novel behaviors, i.e., trained behaviors that the parrots display upon a specific gestural command, but that they had never been asked to repeat before^15,46^. We tested if the parrots were capable of repeating three newly trained behaviors spontaneously from the first repeat trial. The parrots were therefore trained to perform three novel behaviors upon specific commands (see Supplementary Material for description). The training of the new actions occurred after the end of the delayed repeat test and took ~ 45 days. Once the parrot performed the novel behavior associated with the hand signal reliably, we started the test. The first test session started with a trial in which the subject was requested to perform the new behavior followed by the repeat command. Subsequently, the experimenter continued with 2–4 control trials with previously known behaviors and then requested the new behavior again, followed by the repeat command. If the new behavior was repeated correctly in the first trial and second critical trial spontaneously, the parrot was considered successful. And the training for the next novel behavior started the following day.

### Statistical analysis

#### Performance in the repeat test

To test if individuals could remember their own previous behavior, we estimated the probability of responding correctly to the repeat command and compared this to chance-level. We assumed the chance-level to be 1/4 if they chose one of the four behaviors randomly or if they showed a preference for one of these behavior on all trials. To estimate the probability of success we used a Bayesian multilevel model with the following structure:

$$\begin{array}{ll}{\text{response}}_{i}& \sim {\text{binomial}}(1,\hspace{0.25em}{p}_{i})\\ {\text{logit}}({p}_{i})& ={\alpha }_{{\text{individual}}[i]}+{\gamma }_{{\text{behaviour}}[i]}\\ \alpha & \sim {\text{normal}}(\overline{\alpha },\hspace{0.25em}{\sigma }_{\alpha })\\ \overline{\alpha }& \sim {\text{normal}}(-0.5,\hspace{0.25em}1)\\ \gamma & \sim {\text{normal}}(0,\hspace{0.25em}{\sigma }_{\gamma })\\ {\sigma }_{\alpha }& \sim {\text{exponential}}(1)\\ {\sigma }_{\gamma }& \sim {\text{exponential}}(2)\end{array}$$

The prior for the average performance was chosen to center most mass around the chance level of 1/4.

To test performance on the double repeat task the same model was used, with the only modification that the prior for $\overline{\alpha }$ was centered around − 1. Models were fitted using *ulam* from the *rethinking* package^72^ which runs the Stan sampler using the *cmstanr*interface. We ran four chains with 8000 iterations and a 500-iteration warm-up. Rhat and divergence were monitored.

#### Performance in the delayed “repeat” test

To test if and how performance declined with increased delay between the initial behavior and the repeat command a similar Bayesian model was used with a multilevel slope added:

$$\begin{array}{ll}{\text{response}}_{i}& \sim {\text{binomial}}(1,\hspace{0.25em}{p}_{i})\\ {\text{logit}}({p}_{i})& ={\alpha }_{{\text{individual}}[i]}+{\gamma }_{{\text{behaviour}}[i]}+{\beta }_{[i]}*\text{log(time)}\\ \alpha & \sim {\text{normal}}(\overline{\alpha },\hspace{0.25em}{\sigma }_{\alpha })\\ \overline{\alpha }& \sim {\text{normal}}(-1,\hspace{0.25em}1)\\ \gamma & \sim {\text{normal}}(0,\hspace{0.25em}{\sigma }_{\gamma })\\ {\beta }_{[i]}& ={\zeta }_{{\text{individual}}[i]}+{\iota }_{{\text{behaviour}}[i]}\\ \zeta & \sim {\text{normal}}(0,\hspace{0.25em}{\sigma }_{\zeta })\\ \iota & \sim {\text{normal}}(0,\hspace{0.25em}{\sigma }_{\iota })\\ {\sigma }_{\alpha },{\sigma }_{\gamma },{\sigma }_{\zeta },{\sigma }_{\iota }& \sim {\text{exponential}}(2)\end{array}$$

### Ethical approval

All applicable international, national, and institutional guidelines for the care and use of experimental animals were followed. In accordance with the German Animal Welfare Act of 25th May 1998, Section V, Article 7 and the Spanish Animal Welfare Act 32/2007 of 7th November 2007, Preliminary Title, Article 3, the study was classified as non-animal experiment and did not require any approval from a relevant body. The experiments did not require an application to the Animal Ethics Committee of neither Germany nor Spain, as animals participated voluntarily in the experiments and were not affected by them in any way. This article does not contain any studies with human participants performed by any of the authors. The ARRIVE guidelines for the reporting of animal experiments were followed. The informed consent to publish was obtained from all subjects and/or their legal guardian(s) for the publication of identifying information/images.

## Supplementary Information

Supplementary Information 1.

Supplementary Video 1.

Supplementary Video 2.

## Data Availability

All data and code is publicly available on GitHub (https://github.com/simeonqs/Memory_for_own_actions_in_parrots) and permanently stored on Edmond (https://doi.org/10.17617/3.XYSJ66—link will work upon publication). For review data can be accessed on Edmond using the following link: https://edmond.mpdl.mpg.de/privateurl.xhtml?token=2a7cf6ab-7b5f-4732-91ee-d9e1360c9299.
