# Supplementary material for: Memory for own actions in parrots
Source: Sci Rep. 2022 Nov 29;12:20561. doi: 10.1038/s41598-022-25199-x (PMC9709151; doi:10.1038/s41598-022-25199-x)
Supplement: Supplementary file 1 — Supplementary Information 1. [file 41598_2022_25199_MOESM1_ESM.docx]

**Supplementary Materials**

**Housing details**

The parrots were housed in groups of six to eight conspecifics in two different aviaries (1.8 × 3.4 × 3 m each) interconnected by 1 × 1 m windows, which could be closed for separating individuals for testing. Half of the aviary was outdoors so the birds were exposed to natural weather conditions. The other half was covered and lit with Arcadia Zoo Bars (Arcadia 54W freshwater Pro and Arcadia 54W D3 Reptile Lamp) that automatically followed the natural daylight regime.

A large variety of fruit and vegetables was given both in the morning and in the afternoon. Parrots also received Versele Laga Ara seed-mix portioned according to individual body weight together with the fruit in the afternoon.

**Repeat command training**

First, the parrots needed to be trained to perceive the neutral stimulus of a dog whistle as a conditioned stimulus by pairing it with food rewards. The whistle was required for reinforcing desired behaviors in the right moment followed by sunflower seeds as food reward. The frequency of the desired behavior increased by gradual shaping. All parrots were first trained to provide attention to the experimenter. After the parrot had learned to perch calmly in front of the experimenter and attend to her, the four actions were trained and were associated to the different hand gestures (Table SP1).

All subjects were trained using the same training steps:

1. The experimenter reqssuested one of the four trained actions five times in a row by the respective command and then introduced the repeat command expecting the animal to repeat the chosen action by inertia.
2. The experimenter then switched to a different action than the previous and requested the parrot to perform it five times. After that, again the repeat command was given. If the animal did not repeat the second action, the experimenter would request the second action paired with the repeat command until successfully performed by the parrot consistently. When the parrot or if the animal repeated the second behavior correctly, then the experimenter intermixed action 1 and action 2, asking each action 5 times followed by the repeat command. The number of times each action is asked before the repeat command will be decreasing slowly until the animal discriminated correctly and only needed two previous presentations of the two actions before the repeat command to correctly repeat.
3. After the parrots were able to consistently repeat the two actions on command, the experimenter requested a third action just once and then gave the repeat command to test if the parrots were able to repeat a novel action without training. If the animal failed, then the experimenter requested the third action five times and then gave the repeat command again as in step 1. If the animal repeated successfully, the experimenter requested to repeat the three learned actions in random order until the animals discriminated the repetition of those three actions reliably. If the animal failed, the experimenter repeated training sessions with the parrot on repeating the third action until the animal repeated it consistently.
4. Once the animals repeated three actions consistently, the last action was requested followed by the repeat command, to test if the parrots could repeat the fourth “new” behavior instantly. If the animal failed, the experimenter requested the fourth action five times consecutively and then gave the repeat command as stated in step 1. If the animal repeated correctly, the experimenter repeated the four actions in random order until good discrimination is achieved. If the animal failed, the experimenter performed training sessions until the animal repeats the fourth action consistently.
5. The experimenter repeated training sessions on repeating the four actions until the animals passed criterion. The criterion consisted in repeating seven times correctly out of 12 repeat trials. The total number of trials was 28 with 16 control trials (with a 0.25% chance level).

**Table SP1.** Description of the behaviors trained for the repeat test and the delayed repeat test (artwork by Caterina Bonizzato).

| **Behavior** | **Description** | **Hand gesture used (=command)** | **Hand gesture illustrated** |
| --- | --- | --- | --- |
| **Spin** | The parrot spins around its own axis in a clockwise direction | The index finger rotates in a circular movement in clockwise direction over the parrot’s head | 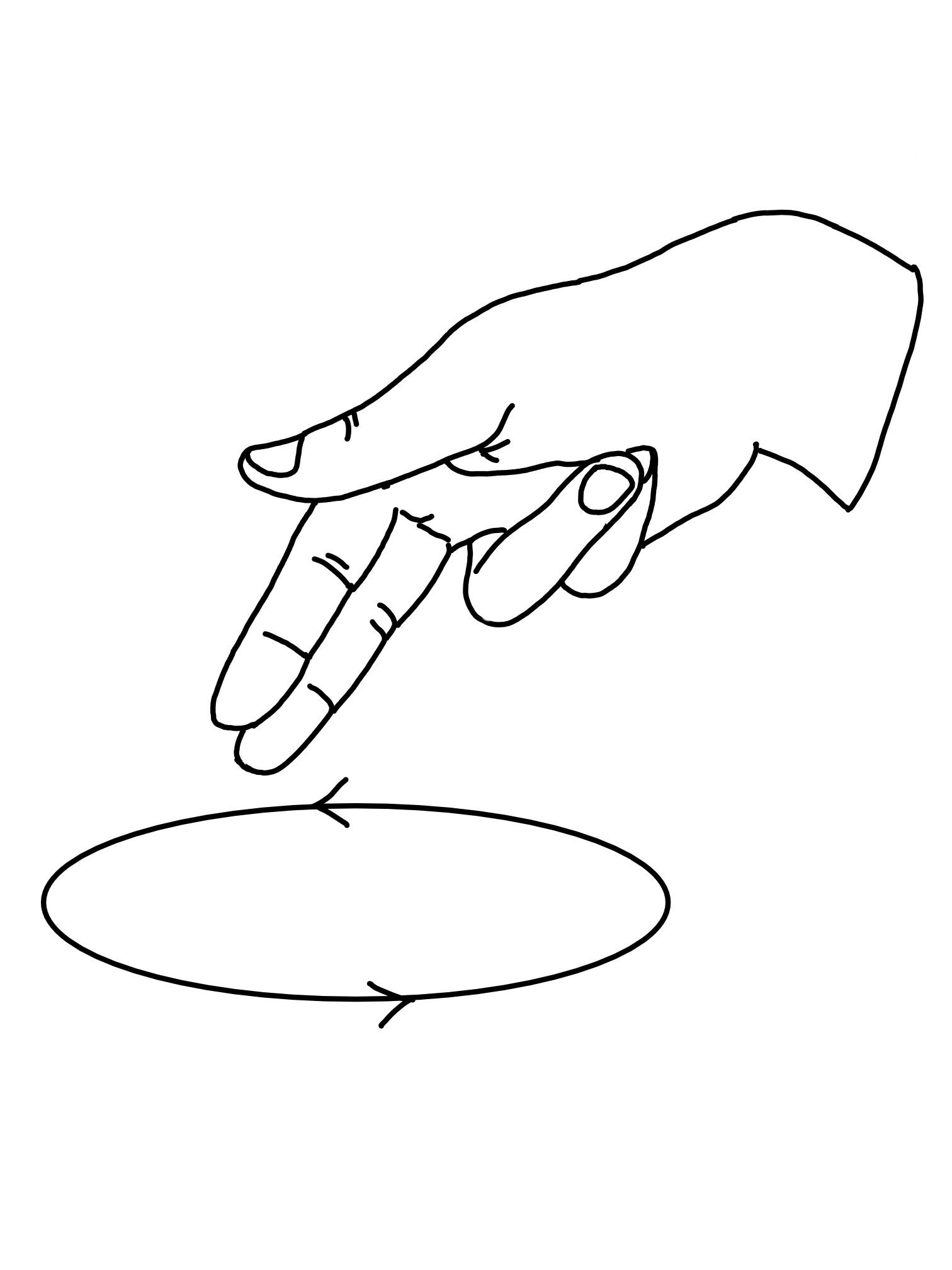 |
| **Wings** | The parrot flaps its wings once | Palm of the hand open and extended moving up and down repeatedly | 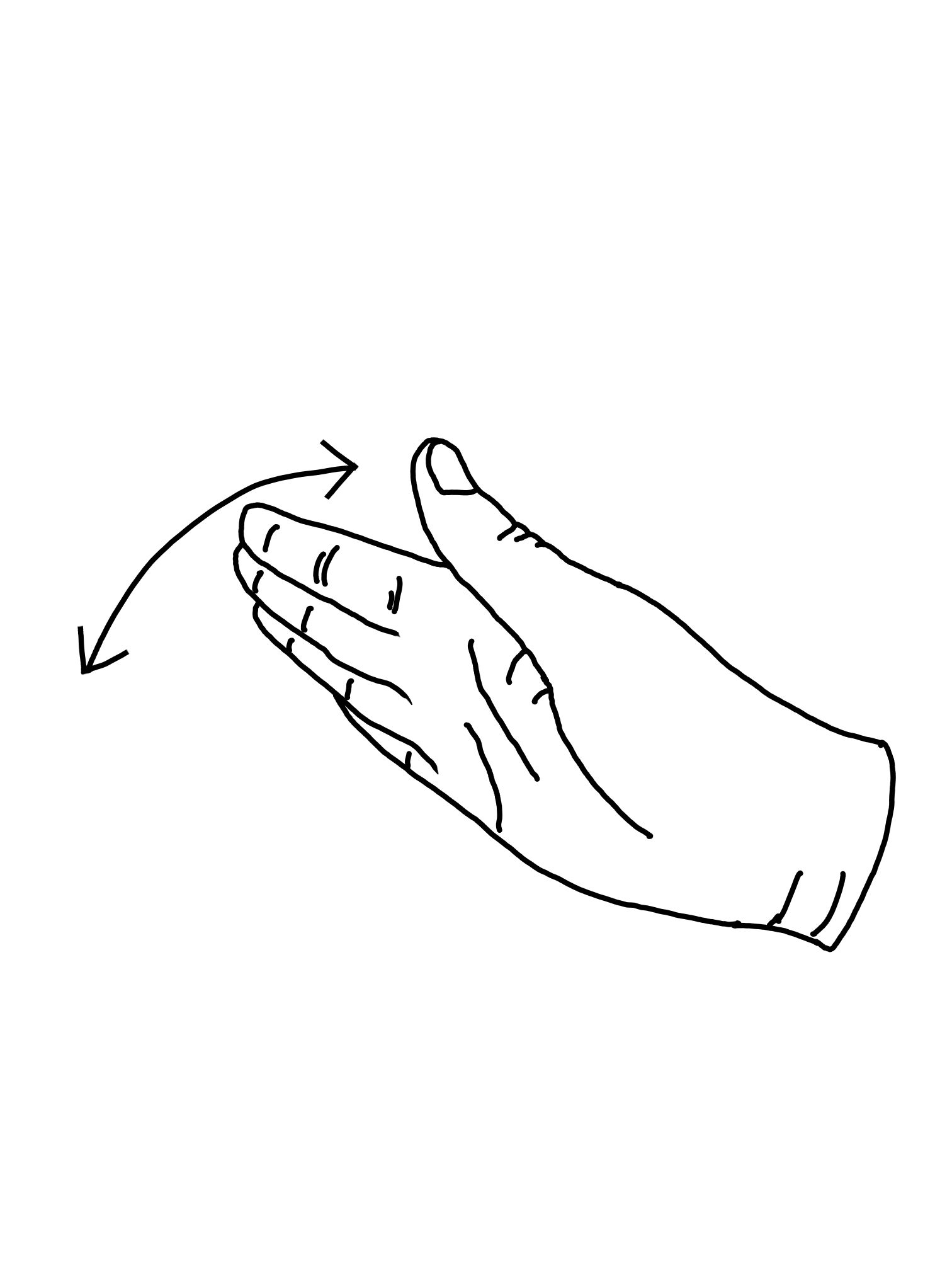 |
| **Lift right leg** | The parrot lifts up its left leg | Index finger in vertical position and remaining fingers closed | 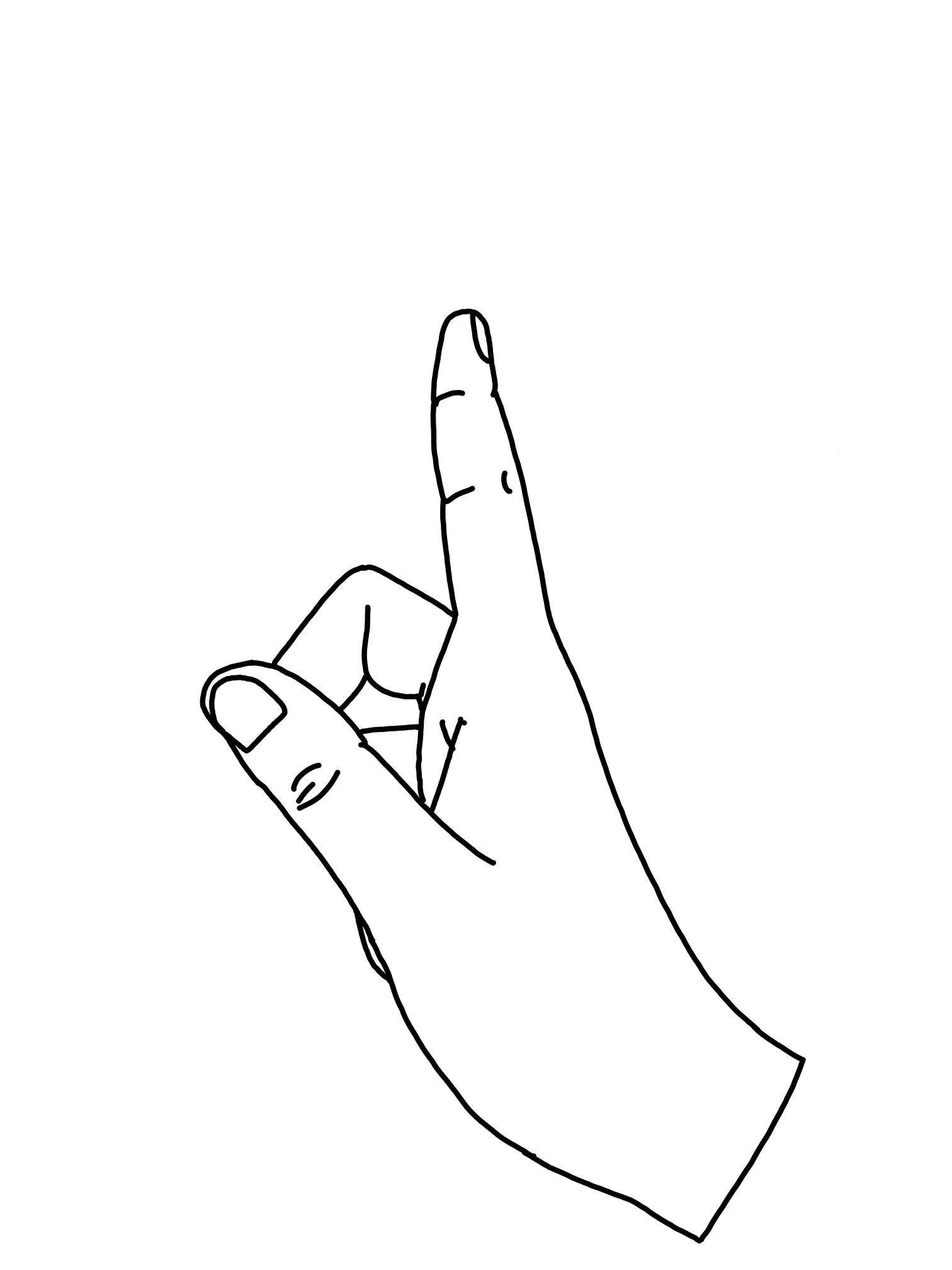 |
| **Head shake** | The parrot shakes its head once | Clenched fist turned horizontally with see-sawing extended thumb and little finger | 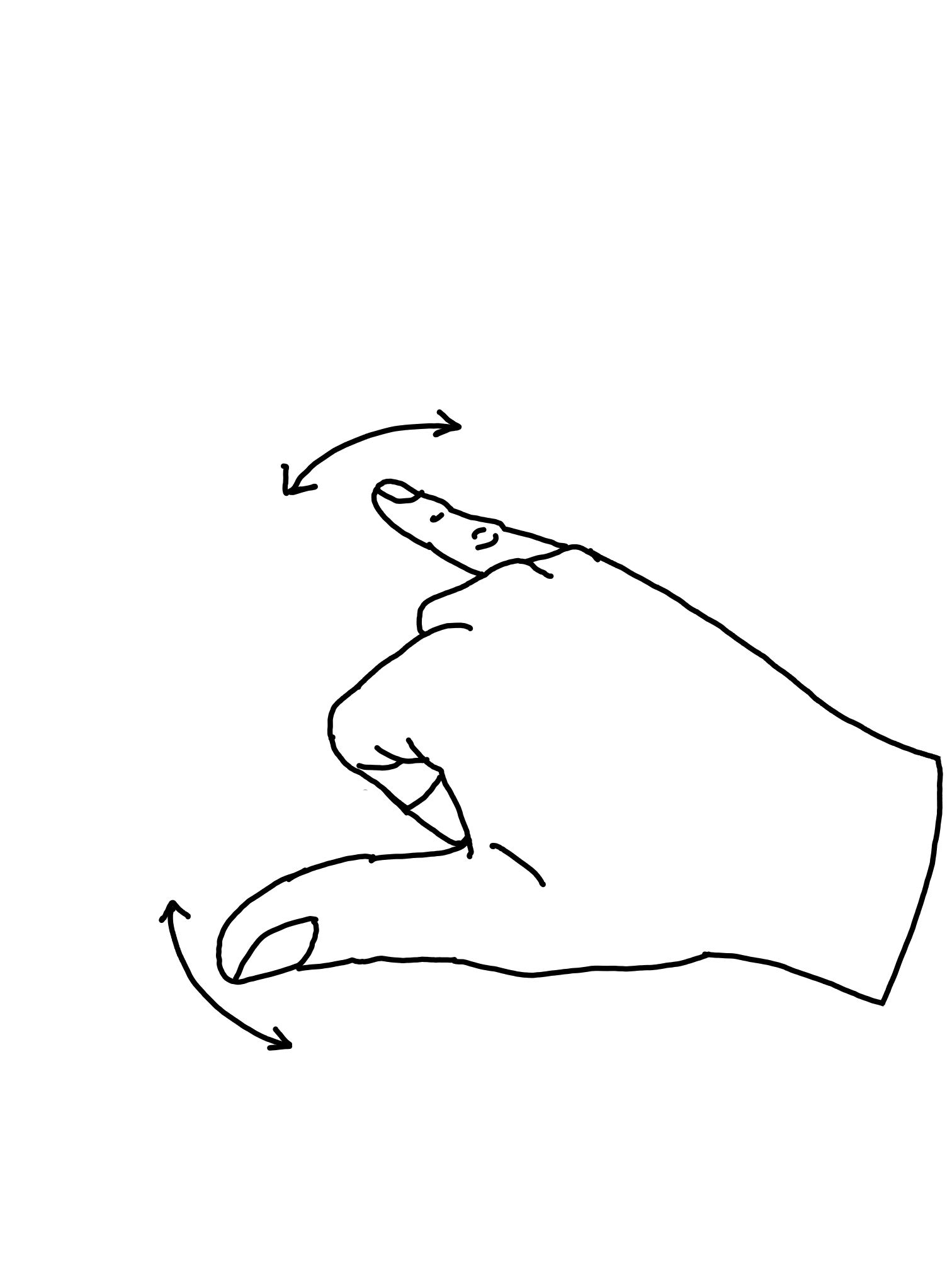 |
| **Repeat** | The parrot repeats the last behavior it had performed upon behavioral command | Index and middle finger extended horizontally and circling | 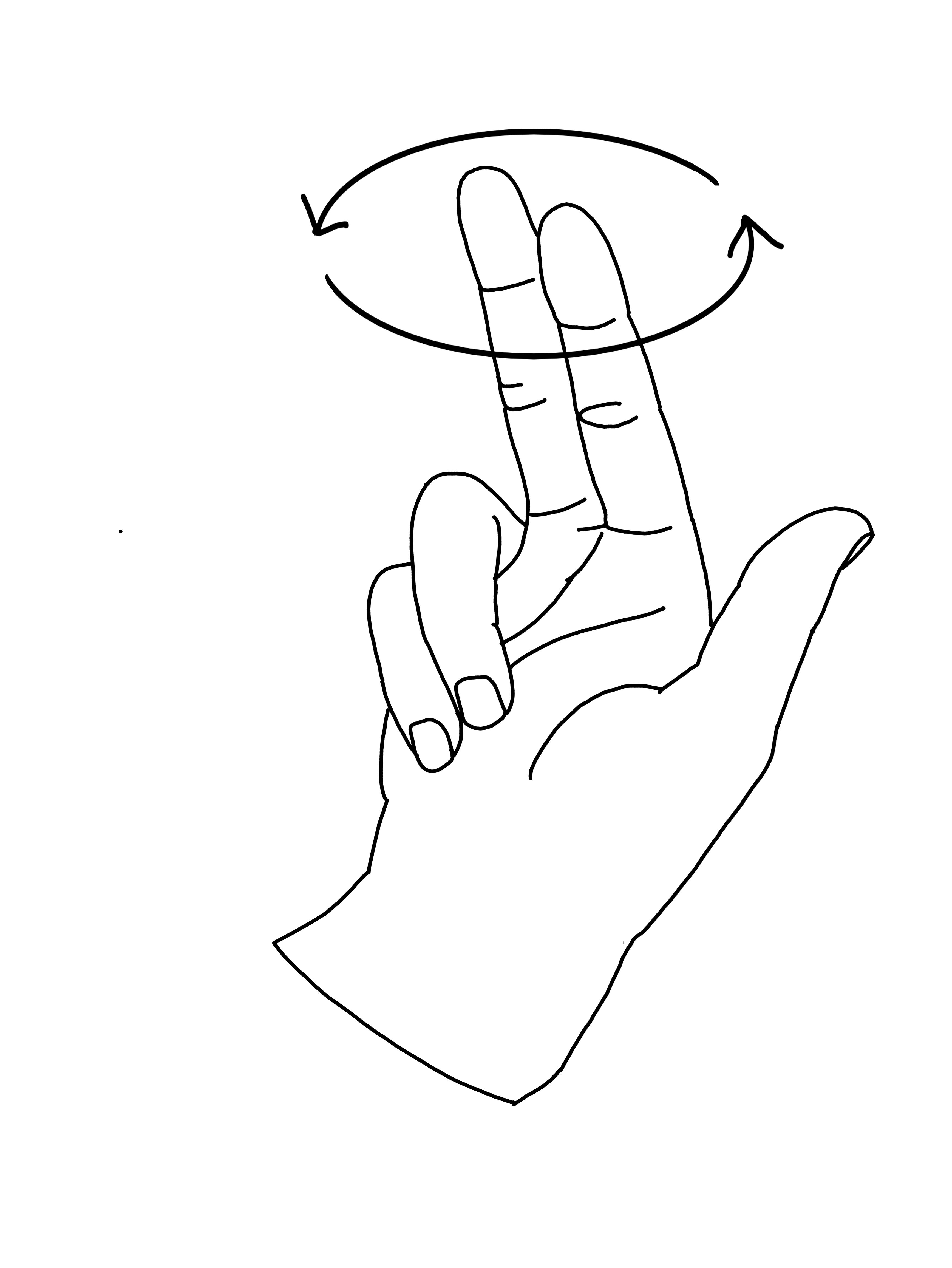 |

**Table SP2.** Description of the subjects

| **Animal ID** | **Species** | **Year of Hatching** | **Gender** |
| --- | --- | --- | --- |
| Charlie | *Ara glaucogularis* | 2014 | Male |
| Mr Huang | *Ara glaucogularis* | 2013 | Male |
| Gargamel | *Ara glaucogularis* | 2012 | Male |
